# Comparative Ser/Thr/Tyr phosphoproteomics between two mycobacterial species: the fast growing *Mycobacterium smegmatis* and the slow growing *Mycobacterium bovis* BCG

**DOI:** 10.3389/fmicb.2015.00237

**Published:** 2015-04-08

**Authors:** Kehilwe C. Nakedi, Andrew J. M. Nel, Shaun Garnett, Jonathan M. Blackburn, Nelson C. Soares

**Affiliations:** Blackburn Lab, Applied Proteomics and Chemical Biology Group, Division of Medical Biochemistry, Institute of Infectious Disease and Molecular Medicine, University of Cape TownCape Town, South Africa

**Keywords:** mycobacteria, phosphoproteome, protein phosphorylation, cell division, growth rate

## Abstract

Ser/Thr/Tyr protein phosphorylation plays a critical role in regulating mycobacterial growth and development. Understanding the mechanistic link between protein phosphorylation signaling network and mycobacterial growth rate requires a global view of the phosphorylation events taking place at a given time under defined conditions. In the present study we employed a phosphopeptide enrichment and high throughput mass spectrometry-based strategy to investigate and qualitatively compare the phosphoproteome of two mycobacterial model organisms: the fast growing *Mycobacterium smegmatis* and the slow growing *Mycobacterium bovis* BCG. Cells were harvested during exponential phase and our analysis detected a total of 185 phospho-sites in *M. smegmatis*, of which 106 were confidently localized [localization probability (LP) = 0.75; PEP = 0.01]. By contrast, in *M. bovis* BCG the phosphoproteome comprised 442 phospho-sites, of which 289 were confidently localized. The percentage distribution of Ser/Thr/Tyr phosphorylation was 39.47, 57.02, and 3.51% for *M. smegmatis* and 35, 61.6, and 3.1% for *M. bovis* BCG. Moreover, our study identified a number of conserved Ser/Thr phosphorylated sites and conserved Tyr phosphorylated sites across different mycobacterial species. Overall a qualitative comparison of the fast and slow growing mycobacteria suggests that the phosphoproteome of *M. smegmatis* is a simpler version of that of *M. bovis* BCG. In particular, *M. bovis* BCG exponential cells exhibited a much more complex and sophisticated protein phosphorylation network regulating important cellular cycle events such as cell wall biosynthesis, elongation, cell division including immediately response to stress. The differences in the two phosphoproteomes are discussed in light of different mycobacterial growth rates.

## Introduction

*Mycobacterium tuberculosis* is the causative agent of tuberculosis (TB), a major health concern worldwide. The current incidence of tuberculosis disease in South Africa is more than 900 cases per 100,000 people per year. Moreover, the World Health Organisation have estimated that roughly one third of the world's population is latently infected with *M. tuberculosis*. The majority of latently infected individuals will remain asymptomatic throughout their lives, with the risk of developing active TB disease from a latent infection being ~10% per lifetime; in HIV positive individuals though, this risk increases to 10% per year (WHO, 2014). This latent infected population is thus a substantial reservoir of potential new TB cases and is therefore a significant public health concern. The ability of mycobacteria to adapt to, and overcome, hostile environmental conditions in order to survive within host cells depends on a fine-tuned and orchestrated series of events (e.g., response to stress; metabolic remodeling; changes in growth rate) that will ultimately define the success of establishing and maintaining long term infection (McKinney et al., 2000; Marrero et al., 2010). *M. tuberculosis* infection is often described by two distinct phases: an active phase, in which the microorganism is thought to be growing at or close to its maximum rate; and latent infection, in which the bacilli are thought to persist in a viable but perhaps more dormant-like state with lower or non-existent growth rate. Current thinking suggests that there is most likely a continuum of states between latent TB infection (LTBI), sub-clinical TB and active TB disease, but to date no *M. tuberculosis* bacilli have been observed in LTBI individuals, so the exact physiological state of *M. tuberculosis* during a latent infection remains unknown.

Alongside the increasing number of new TB infections there is another matter of great concern, which is the emergence and spread of multi- and extensively drug resistant *M. tuberculosis* strains. Here unique growth related mechanisms of *mycobacteria* which facilitate adaptation to different adverse micro-environments are thought to play an important role in the mechanisms of drug tolerance and acquired resistance that are observed during infection (Corper and Cohn, 1933; Wayne and Hayes, 1996). For instance, a recent study showed that diverse growth-limiting stresses trigger a common signal transduction pathway in *M. tuberculosis* that induces triglyceride synthesis, which is associated with slowing down of growth and reduced antibiotic efficacy (Baek et al., 2011). Therefore, further investigation of the signaling pathways which regulate mycobacterial growth rate might reveal important information regarding the capacity of *M. tuberculosis* to adapt to its environment and in particular how this relates to drug tolerance and to the ability to establish infection.

In *M. tuberculosis*, Ser/Thr phosphorylation is essential for growth and environmental adaptation (as reviewed by Kieser and Rubin, 2014). For instance, the Ser/Thr protein kinases (STPKs) PknA and PknB are essential for growth of *M. tuberculosis* in culture, indicating that phosphorylation plays a pivotal role in the survival of this bacterium (Sassetti et al., 2003; Kang et al., 2005; Fernandez et al., 2006; Molle and Kremer, 2010; Kusebauch et al., 2014). These STPKs are encoded by an operon which regulates genes involved in cell shape determination, cell wall synthesis, and cell division (Deol et al., 2005; Kang et al., 2005; Kusebauch et al., 2014). In addition to PknA and PknB, another group of STPKs comprised of PknG, PknL, and PknF appear to be involved in different aspects of *M. tuberculosis* growth regulation (Cowley et al., 2004; Deol et al., 2005; Canova et al., 2008). In support of the likely important role played by STPKs in *M. tuberculosis*, a mass spectrometry-based phosphoproteomic study identified more than 500 Ser/Thr phosphorylation sites on 300 proteins in *M. tuberculosis* (Prisic et al., 2010) and, more recently, a complementary study detected a number of Tyr phosphorylated proteins in *M. tuberculosis* (Kusebauch et al., 2014). Notably though, the physiological significance of these findings remains largely unexplored.

In the past years, the use of mycobacterial models such as *M. smegmatis* and *M. bovis* BCG have significantly contributed to our current understanding of *M. tuberculosis* biology and environmental adaptation (as reviewed by Shiloh and Champion, 2010). *M. bovis* BCG is an attenuated bovine tuberculosis bacillus by a serial passage in the laboratory (Calmette et al., 1921). This mycobacterium is a particular convenient model in part due to it is slow growth rate similar to that observed in *M. tuberculosis*. On other hand *M smegmatis* is a fast growing mycobacterial species (with a doubling time of approximately 4 h) that has been widely used to investigate different aspects of mycobacterial physiology (Barry, 2001; Reyrat and Kahn, 2001; Danilchanka et al., 2008). As part of a concerted program to associate protein phosphorylation in mycobacteria with subsequent macromolecular events which determine growth rate and eventual environmental adaption, we have carried out a phosphopeptide enrichment and high throughput mass spectrometry-based study to investigate and compare the phosphoproteome of two model mycobacterial organisms—the fast growing *M. smegmatis* and the slow growing *M. bovis* BCG—our goal being to begin to elucidate the phosphorylation events and subsequent signal transduction pathways coordinating differential mycobacterial growth rates, which may in due course lead to important insights into *M. tuberculosis* physiology during active and latent infection—including aspects of slow growth associated drug resistance.

## Materials and methods

### Bacterial strains

Wild type strains of *M. smegmatis* mc^2^155 and *M. bovis* BCG Pasteur strain 1173P2 were grown in 7H9 Middlebrook (BD, Maryland, USA) broth supplemented with 0.05% Tween 80, OADC (Becton Dickinson) and 0.2% glycerol (v/v). Cells were grown at 37°C with continuous agitation (120 rpm).

### Protein extraction

Cells were harvested during the exponential phase (OD_600_ ~1.2 and 0.6 for *M. smegmatis* and *M. bovis* BCG, respectively) by centrifugation at 4000 g for 15 min at 4°C, washed twice with Phosphate Buffered Saline pH (7.5) (PBS). Cells were snap frozen in liquid nitrogen and stored at -80°C until needed. Frozen pellets were suspended in 800 μl lysis buffer [500 mM Tris-HCl, 0.1% (w/v) SDS, 0.15% sodium deoxycolate, 1× protease inhibitor cocktail, 1× phosphatase inhibitor cocktail (Roche, Mannheim Germany) and 50 μg/ml lysozyme (Repaske, 1956)]. Cells were disrupted by sonication at maximum power for six cycles of 30 s, with 1 min cooling on ice between cycles. Cellular debris was removed by centrifugation at 4000 g for 5 min and the lysate filtered through 20 μm pore size low-protein binding filters (Merck, NJ, USA). Proteins were precipitated using the chloroform-methanol precipitation method as previously described (Wessel and Flügge, 1984). The pellet was re-suspended in urea buffer (6 M urea, 2 M thiourea and 10 mM Tris-HCl, pH 8). Protein concentration was determined using a modified Bradford assay as described by Ramagli (1999).

### In-solution trypsin digestion

Five milligram of total protein was reduced with 1 mM DTT for 1 h with agitation and then alkylated with 5.5 mM IAA for 1 h in the dark. Proteins were pre-digested with Lysyl Endopeptidase LysC (Waco, Neuss, Germany) at room temperature for 3 h and diluted 4× with HPLC grade water before adding sequencing grade modified trypsin (Promega, Madison, USA) (1:100 w/w). Proteolysis was carried out at room temperature for 14 h with agitation at 30 rpm. Proteolysis was quenched by addition of trifluoroacetic acid (TFA) (Sigma Aldrich, St Louis, USA).

### Phosphopeptide enrichment with TiO_2_ chromatography

Acetonitrile (ACN) (Sigma Aldrich, St Louis, USA) at a final concentration of 30% was added to the tryptic peptides and the pH was adjusted to 2. Ten milligram of Titasphere TiO2 beads (MZ Analysentechnik, Mainz, Germany) in loading buffer [30 mg/ml 2,5 dihydrobenzoic acid (Sigma Aldrich, St Louis, USA), 80% ACN] were added to the sample and incubated at room temperature with rotation for 1 h. The beads were pelleted and the decanted supernatant was further incubated with a fresh batch of 5 mg of beads for 30 min. This step was repeated one further time, giving three fractions of enriched phosphoproteins bound to beads in total. Phosphopeptides bound to the beads were washed vigorously with shaking for 10 min in 1 ml of wash buffer 1 (30% acetonitrile, 3% trifluoroacetic acid) followed by an additional 10 min of vigorous wash with wash buffer 2 (80% acetonitrile, 0.1% trifluoroacetic acid). Phosphopeptides were loaded onto C8 stage tip, washed with wash buffer 2 and then eluted with 3 × 50 μl Elution buffer [40% Mass-spec grade NH_4_OH (aq, 25% NH_3_; Sigma-Aldrich, St Louis, USA), 60% acetonitrile (pH 10.5 or higher)]. Solvent was removed in a speed drying vacuum at room temperature and peptides were resuspended in 20 μl Solvent A (2% ACN, 0.1% formic acid).

### LC/MS/MS analysis

Liquid chromatography separation was carried out using a precolumn (100 μm ID × 20 mm) connected to an analytical column (75 μm × 500 mm) packed with C18 Luna 5 μ 100 Å beads (phenomenex 04A-5452). The columns were connected to an Easy n-LC II system (Proxeon). 5 μl of resuspended phosphopeptides were loaded onto the column with starting mobile phase of 2% ACN, 0.1% formic acid. Peptides were eluted with the following gradient 5% ACN to 35% ACN for 120 min the up to 80% ACN over 5 min staying at 80% ACN for 10 min, this was followed by a column wash of 7 to 80% gradient for 30 min, and a column equilibration at 2% ACN for 2 min. The flow rate was held at 300 nL/min.

Mass spectra were acquired on an Orbitrap Q Exactive mass spectrometer in a data-dependent manner, with automatic switching between MS and MS/MS scans using a “Top-10” method. MS spectra were acquired at a resolution of 70,000 with a target value of 3 × 10^6^ ions or a maximum injection time of 250 ms. Peptide fragmentation was performed via higher-energy collision dissociation (HCD) with the energy set at 25 NCE. Intensity threshold for ions selection was set at 1.7e4 with charge exclusion of *z* = 1 and *z* > 5. The MS/MS spectra were acquired at a resolution of 17,500, with a target value of 2 × 10^5^ ions or a maximum injection time of 120 ms. The scan range was limited to 300 to 1750 m/z and the isolation window at 4.0 m/z.

### Data processing

Maxquant software package version 1.5.0.3 with integrated Andromeda search engine was used to analyse the raw MS spectra acquired. *M. smegmatis* (taxonomy 246196) and *M. bovis* BCG Pasteur (taxonomy 410289) databases obtained from Uniprot were used as the target-decoy databases for the Andromeda search. LysC and Trypsin were defined as proteases and two missed cleavages were allowed. Phosphorylation on serine, threonine, and tyrosine residues, oxidation of methionines and N-terminal acetylation were specified as variable modifications. Carbamidomethylation on cysteines was defined as a fixed modification. We allowed a FTMS MS/MS match tolerance of 20 ppm. False discovery rate was set at 1% for identification of peptides and proteins without re-quantifying. Stringent filtering criteria were employed to validate phospho sites. Phosphopeptides were considered as high confidence phosphorylation events and taken for further analysis only if they had a localization probability of >0.75, had a posterior error probability (PEP) score of <0.001. We further manually validated the phospho sites on the Maxquant viewer for good b- and y- ion series coverage.

### Bioinformatics

Predicted Gene Ontology (GO) cellular functions of identified phosphoproteins with high confidence from two mycobacterium strains were obtained from The Database for Annotation, Visualization and Integrated Discovery (DAVID, v6.7) and were grouped into functional categories. To compare the identified phosphoproteomes of these two mycobacterial species, we identified homologs of the *M. smegmatis* phosphorylated proteins in *M. bovis* BCG using the University of Cape Town's Computational Biology online bioinformatics tool found at http://galaxy-fen.uct.ac.za/root. For identification of tyrosine-phosphorylation site motifs, we used the Motif-X algorithm (Schwartz, 2005). We defined a sequence window of +/- 10 amino acids on each side of the tyrosine site and generated the sequence logo by Phosphosite logo generator using the algorithm PSP production (Cell signaling Technology). We aligned genomic sequences of both strains using the online tool, obtained from http://www.ebi.ac.uk/Tools/msa/clustalw2/ and respective fasta files were obtained from Uniprot.

## Results and discussion

There is increasing evidence indicating that protein phosphorylation plays an essential role during mycobacterial cell division and environmental adaptation. Understanding the mechanistic connection between such regulatory signaling networks and mycobacterial growth rate requires a global view of the phosphorylation events taking place at a given time under defined conditions. Here, we have carried out a comparative phosphoproteomic analysis of two mycobacterial strains—one fast growing (*M. smegmatis*), the other slow growing (*M. bovis* BCG). Our analysis included three rounds of TiO_2_ chromatography to enrich phosphorylated peptides (see Supplementary Figure S1), followed by subsequent analysis of phosphorylation-events using liquid chromatography coupled with state-of-the-art high resolution tandem mass spectrometry (LC/MS/MS) (Supplementary Figure S2a and S2b), in order to compare the phosphoproteomes of these two model mycobacterium species during exponential growth.

**Figure 1:**
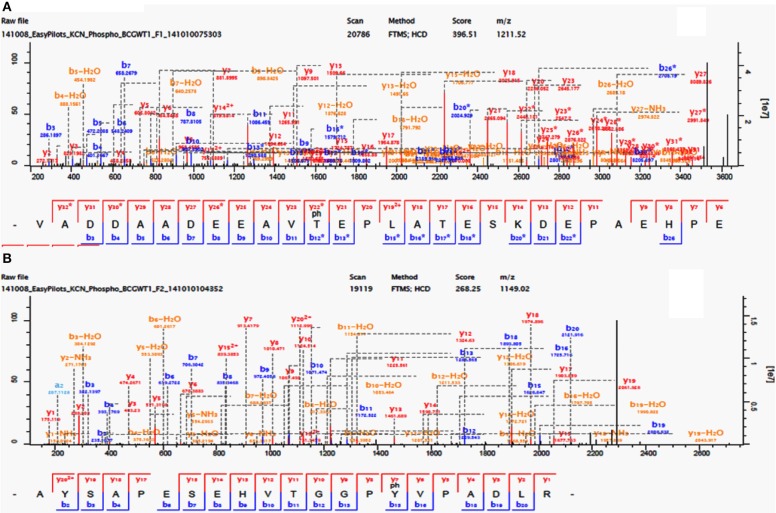
**Phosphorylation of *M. bovis* BCG Cell division FtsQ (Thr_24_) and probable conserved protein membrane mmpS3 (Tyr_70_). (A)** Identification of phosphorylated residue by mass spectrometry. Fragmentation spectra for modified peptide bearing the phosphorylated Thr_24_. **(B)** Fragmentation spectra for modified peptide bearing the phosphorylated Tyr_70_.

In this study we considered all phosphorylation-events that were detected in any of the biological replicates, and only confidently localized phospho-sites (*p*-sites) (Figure 1), were considered for qualitative comparative analysis and further discussion. In the two *M. bovis* BCG replicates, we detected a total of 442 *p*-sites of which 289 were confidently localized (Localization probability (LP) = 0.75; PEP = 0.01) and 169/289 had a LP = 0.99 (Supplementary Table S1A and Figure S2c). We identified 88,822 MS/MS spectra corresponding to 7784 non-redundant peptide sequences (Supplementary Figure S2d) and 1765 protein groups (402 were identified by a single peptide) (Supplementary Table S1A). The estimated false discovery rate (FDR) was 0.32 at the peptide level, 0.30 at the modification level, and 1.03 at the protein level. Our initial analysis of two biological replicates of *M. smegmatis* revealed considerable differences in the number of identified p-sites between the two Mycobacterial species, 77 *p*-sites for *M. smegmatis* (Supplementary Figure S2e) compared to 289 for *M. bovis* BCG. To verify that these differences observed were biological, we further analyzed three additional biological replicates for *M. smegmatis* (Supplementary Table S1B). Phosphoproteomic analysis of five biological replicates of *M. smegmatis* protein extracts resulted in identification of 180, 396 MS/MS spectra, corresponding to 16, 185 non-redundant peptide sequences (Supplementary Figure S2f) and 2, 462 protein groups (464 were identified by a single peptide) (Supplementary Table S1B). The estimated false discovery rate (FDR) was 0.22 at the peptide level, 0.21 at the modification level, and 0.98 at the protein group level. We detected a total of 185 phospho-sites in *M. smegmatis*, of which 106 were confidently localized (LP = 0.75; PEP = 0.01) and 64/106 had a LP = 0.99 (Supplementary Table S1B). In detail, for *M. bovis* BCG, we detected 289 *p*-sites on 203 phoshoproteins: 35.3% on serine (pSer), 61.6% on threonine (pThr) and 3.1% tyrosine (pTyr). For *M. smegmatis* we detected 106 *p*-sites on 76 phosphoproteins: 39.47% on serine (pSer), 57.02% on threonine (pThr) and 3.51% on tyrosine (pTyr). Both phosphoproteomes were biased toward Thr compared with Ser (57–61%; 41–35%), which agrees with previous reports on *M. tuberculosis* H37Rv (Prisic et al., 2010). Importantly, the percentage of Tyr phosphorylation in *M. bovis* BCG was closer to that reported in *M. tuberculosis* (Kusebauch et al., 2014). Although it had previously been suggested that Tyr phosphorylation was non-existent within mycobacterial species, it was recently confirmed that Tyr phosphorylation does in fact occur on a number of diverse *M. tuberculosis* proteins (Kusebauch et al., 2014). Here, we have confidently identified nine Tyr p-sites in eight proteins in *M. bovis* BCG (Table 1, Figure 1B and Supplementary Table S1A) and four in *M. smegmatis*, supporting earlier suggestions that phosphorylation on Tyr residues occur in different mycobacterial species (Kusebauch et al., 2014).

**Table 1:** **List of Tyr phosphorylated proteins/sites identified in *M. bovis* BCG and *M. smegmatis***.

**Acc. number^a^**	**Protein description**	**Phosphorylated peptide sequence and phosphorylated site**	**Position of the phosphorylated Tyr**
A1KEI8	Uncharacterized protein FHA domain-containing protein	HPGQGDYPEQIG**Y(1)**^b^PDQGGYPEQR	215^*^
		GGYPPETGGYPPQPG**Y(1)**PRPR	232^*^
A1KFR2	60kDa chaperonin 1	VAQIRQEIENSDSD**Y(0.919)**DREK	358^*^
A1KFP3	Uncharacterized protein Hydrolase domain-containing protein	GLAEGPLIAGGHS**Y(1)**GGR	99
A1KI63	Uncharacterized protein	SA**Y(0.913)**PDGIADHDRPLAPR	8
A1KKP0	Probable isocitrate lyase aceA	MGIEAI**Y(0.998)**LGGWATSAK	104
A1KKP0	Probable conserved membrane protein mmpS3	AYSAPESEHVTGGP**Y(1)**VPADLR	70^*^
A1KKR9	Uncharacterized protein CYTH-like domain containing protein	**Y(0.847)**TAATGADNVSQEAK	428
A1KPH7	Conserved hypothetical mce associated protein	RDCASVMV**Y(0.973)**LNRTVTDK	122
^^^A0QWX0	Carbohydrate kinase FGGY	**Y(1)**NYDTLAGR	1
^^^A0QQ14	Carbohydrate kinase FGG	ISAW**Y(0.968)**VER	5
^^^A0QSS3	10 kDa chaperonin	**Y(1**)GGTEIK	1
^^^A0QQU5	60 kDa chaperonin 1	AEIENSDSD**Y(0.915)**DREK	10

* *Tyr phosphorylated sites that were previously identified in M. tuberculosis H37Rv (Kusebauch et al., 2014)*.

^ *Tyr phosphorylated sites in M. smegmatis*.

a *Uniprot protein accession number*.

b *Phosphorylated Tyr (Y) and respective Localization probability*.

The differences between the compared phosphoproteomes compelled us to investigate whether some of these dissimilarities could be explained by genomic events rather than post-translational control. The multiple sequence alignment of 130 selected *M. bovis* BCG phosphoproteins with their respective *M. smegmatis* orthologs (Supplementary S3A) revealed that from 197 *M. bovis* BCG Ser/Thr/Tyr phosphorylated sites: 12 are conserved across the two mycobacterial species and were found to be phosphorylated in both species, while 94 conserved Ser/Thr/Tyr residues were found to be phosphorylated in *M. bovis* BCG only. Furthermore, whereas 91 *M. bovis* BCG phosphorylated residues were aligned with a different non-phosphorylated *M. smegmatis* residue, 72 of these were aligned with a non-phosphorylated amino acid (Supplementary S3A) and 19 were aligned with different non-phosphorylated Ser/Thr/Tyr residue. Conversely, the multiple sequence alignment of 64 *M. smegmatis* phosphoproteins with their respective *M. bovis* BCG orthologs showed that besides the 12 conserved Ser/Thr/Tyr residues phosphorylated in both species, 31 conserved residues were found to be phosphorylated in *M. smegmatis* only. In this case, while 43 *M. smegmatis* phosphorylated residues were aligned with a different non-phosphorylated *M. bovis* BCG amino-acid, 36 of these were aligned with a non-phosphorylated amino acid residue (Supplementary S3B) and seven were aligned with different non-phosphorylated Ser/Thr/Tyr residue (Supplementary S3B). These results point out that some of differences observed between the two phosphoproteomes can be explained by the absence of the corresponding amino acid residue, indicating that during exponential growth phase these two mycobacterial species present an inherently different sub-set of Ser/Thr/Tyr kinase substrates. Additionally, there are some interesting examples in which orthologous proteins were phosphorylated at different *p*-sites. This suggests that kinase specificities for a substrate could be intimately related with the actual site of phosphorylation. Finally, it is notable that occasions the Ser/Thr/Tyr residue was aligned with different Ser/Thr/Tyr residue (in most cases S for T and vice versa) in some punctual situation the respective residue was phosphorylated (e.g., PknB) but for the majority of the cases these were aligned with non-phosphorylated Ser/Thr/Tyr residue. This intriguing observation leaves open the possibility that Ser/Thr exchange could be a result of an evolutionary processes/environmental adaptation, in which the replacement for the respective residue would probably favor site phosphorylation and therefore the gain of an additional mechanism of protein functional regulation. Although speculative, it would be interesting to explore further in which conditions these sub set of Ser/Thr/Tyr sites are phosphoryalated.

Finally, the number of phosphoproteins/sites identified in *M. smegmatis* is comparable to those reported in other soil bacteria, e.g., *E. coli* (Macek et al., 2008; Soares et al., 2013), *Bacillus subtilis* (Shi et al., 2014) and *Pseudomonas putida* (Ravichandran et al., 2009), as well as in some pathogenic bacteria such as *Pseudomonas aeruginosa* (Ravichandran et al., 2009), *Streptococcus pneumonia* (Sun et al., 2009), *Helicobacter pylori* (Ge and Shan, 2011), and *Klebsiella pneumonia* (Lin et al., 2009). Whereas, the number of phosphorylated Ser/Thr/Tyr detected in *M. bovis* BCG phosphoproteome is comparable to that described in *M. tuberculosis* H37Rv (Prisic et al., 2010). It is of particular interest that the *M. bovis* BCG phosphoproteome shows a number of phosphoproteins/sites that are orthologous to those reported in *M. tuberculosis* H37Rv (Prisic et al., 2010) but also a number that are not conserved. Recently a comparison between the Ser/Thr/Tyr phosphoproteomes of *Acinetobacter baumannii* reference strain (ATCC17978) and a highly invasive, multidrug resistant clone (AbH12O-A2) demonstrated that, during stationary phase, the multidrug isolate showed twice as many phosphorylation-events as the reference strain (Soares et al., 2014). In contrast to reports on *Pseudomonas* species (Ravichandran et al., 2009), our current study supports the notion that bacteria within the same genus/species may utilize differing numbers of phosphoproteins.

### Phosphoproteomic analysis reveals conserved Ser/Thr/Tyr phosphorylated sites across mycobacterial species

#### Conserved Ser/Thr phosphorylated sites

Macek et al. (2008) reported evidence of a possible high degree of conservation within potential bacterial phospho-sites although, as noted by those authors, the conservation of residues does not mean that they are phosphorylated in all species. In fact, as the number of bacterial phosphoproteomic studies increases, it is becoming clearer that the degree of conserved phospho-sites among bacterial species is rather limited and certainly lower than reported within eukaryotic phosphoproteomes (e.g., Freschi et al., 2014). Here, a comparison between the *M. smegmatis*, *M. bovis* BCG, and *M. tuberculosis* H37Rv (Prisic et al., 2010; Kusebauch et al., 2014) phosphoproteomes revealed that these three mycobacterial species share a number of conserved phosphorylated sites (Table 2). Interestingly, we found that *M. bovis* BCG and *M. tuberculosis* H37Rv phosphoproteomes share at least 32 Ser/Thr conserved phospho-sites on 27 proteins, of which three were conserved in all three species (Table 2). As pointed out by Freschi et al. (2014), phosphorylation sites that are phosphorylated in different species are more likely to be functional and this conservation criterion could be used to prioritize phosphorylation events for additional characterization.

**Table 2:** **Summary of the phosphorylated sites found in more than one Mycobacterial species**.

**Acc. number^a^**	**Protein description**	***M. bovis* BCG**	***M. smegmatis***	Mtb H37Rv^*^
P65727	Ser/Thr-protein kinase PknA	Ser^b^_309_	Ser_310_	Ser_309_
		Ser_316_	Thr_316_	-
		Ser_299_; Thr_301_;Thr_302_	-	Ambiguous Residues _299−302_
		Thr_224_		Thr_224_
P0A5S5	Ser/Thr-protein kinase PknB	Thr_173_	Thr_173_	Thr_173_
		Thr_171_	Thr_171_	-
Q02251	Mycocerosic acid synthase	Ser_2111_	-	Ser_2111_
Q7TVL9	Possible acyltransferase	Ser_230_	-	Ser_230_
P64169	Cell division protein FtsQ	Thr_24_	-	Thr_24_
Q7TY31	Conserved alanine and glycine and valine rich	Thr_232_	-	Thr_232_
Q7U2K5	Possible conserved transmembrane transport protein MMPL3	Thr_910_	-	Thr_910_
		Thr_893_	-	Thr_893_
Q7U2N3	Probable conserved MCE associated membrane protein	Thr_16_	-	Thr_16_
Q7VEQ4	L-aspargine permease 1	Thr_474_	-	Thr_474_
Q7U280	Isoniazid inductible gene protein	Ser_62_	-	Ser_62_
P65379	Putative membrane protein mmpS3	Ser_58_	-	Ambiguous residues _58−66_
		Thr_66_	-	Thr_66_
		Thr_47_	-	Thr_47_
		Thr_50_	-	Thr_50_
P0A515	Guanylate Kinase	Thr_9_	-	Thr_9_
Q7TXB8	Phosphoglucomutase PGMA	Ser_147_	Ser_147_	Ser_147_
		-	Ser_142_	Ambiguous residues _135−152_
P0A521	60 kDa chaperonin 2	Thr_146_	-	Thr_146_
P45811	30S ribosomal protein S4	Thr_147_	-	Thr_147_
Q7U046	Probable lipase LIPH	Ser_165_	-	Ser_165_
Q7TXZ1	Cell division transmembrane protein FTSK	Thr_642_	-	Thr_642_
P66947	Probable acetolactate synthase	Thr_5_	-	Thr_5_
P66843	Signal recognition particle receptor FtsY	Thr_72_	-	Thr_72_
P66890	Sec-independent protein translocase TatA	Thr_60_	-	Thr_60_
		Thr_78_	-	Thr_78_
P0A549	Chaperone protein DnaJ1	Thr_120_	-	Thr_120_
Q7TVL6	Possible phosphotransferase	Ser_250_	-	Ser_250_
P6387	Chaperone protein ClpB	Thr_79_	-	Thr_79_
P63857	Cytochrome c oxidase subunit 3	Thr_7_	-	Ambiguous residues _2−14_
		Thr_13_	Thr_13_	Ambiguous residues _2−14_
Q7TYA1	Export membrane protein SecF	Ser_396_	-	Ambiguous residues _372−407_
Q7U241	Probale Phosphoribosylglycinamide	Thr_206_	-	Thr_206_
Q7TVC7	Probable peptidoglycan hydrolase	Thr_43_	-	Thr_43_
Q7TTR2	Long-chain-fatty-acid-AMP FadD32	Thr_552_	-	Thr_552_
P0A611	Possible transmembrane cation	Ser_268_	-	Ser_268_
POA611	Single-stranded DNA-binding protein SSB	Ser_152_	-	Ambiguous residues _123−154_
P0A727	Transcriptional regulatory protein PrrA	Thr_6_	-	Thr_6_
P65379	Putative membrane protein mmpS3	Tyr_70_	-	Tyr_70_
Q7U303	Conserved protein with fha domain	Tyr_232_	-	Tyr_232_
		Tyr_215_	-	Tyr_215_
P0A521	60kDa Chaperonin 2	Tyr_358_		Tyr_358_

* *Ser/Thr phosphorylated sites that were previously identified in M. tuberculosis H37Rv (Prisic et al., 2010)*.

a *Uniprot protein accession number*.

b *Ser/Thr residue found phosphorylated in the indicated Mycobacterial species*.

*−, Not detected*.

In the present study we have focused in particular on the STPKs PknB and PknA that have known or predicted functions in cell wall generation and growth in *M. smegmatis*, *M. bovis* BCG, and *M. tuberculosis* (Gee et al., 2012; Kusebauch et al., 2014). We found PknB to be phosphorylated in Thr_173_ in all three species and in Thr_171_ in *M. smegmatis* and *M. bovis* BCG (Table 2). Previous *in vitro* assays demonstrated that both Thr_173_ and Thr_171_ are conserved auto-phosphorylated residues that lie in the activation loop of PknB (Boitel et al., 2003). Additionally, a *M. tuberculosis* double mutant Thr_171_/Thr_173_ was 300-fold less active than respective wild-type PnkB, suggesting a combined effect of both Thr_171_ and Thr_173_ residues on kinase activity. Subsequent studies confirmed that the mutation of these residues had a strong effect not only on PknB kinase activity but also in the process of activation loop-mediated recruitment of its substrates (Villarino et al., 2005). Here we have provided evidence that Thr_173_ and Thr_171_ phosphorylation both occur *in vivo* during the exponential phase, at which PknB is most abundant and is thought be at its maximum activity. Thus, our data reinforces a previous hypothesis suggesting that *in vivo* this enzyme is regulated through an auto-phosphorylation mechanism involving the phosphorylation state of *both* Thr_173_ and Thr_171_.

Another proposed mechanism of PknB regulation relates to the maintenance of an inactive state *via* the interaction of the juxtamembrane region with the kinase domain. In this model, the auto-phosphorylation of specific residues in the juxtamembrane sequences releases the inhibition by making the sequence available for further interactions with domains of target proteins (Wybenga-Groot et al., 2001). However, previously it was not clear whether Thr_294_ and/or Thr_309_ were the target residues involved so it is notable that our data clearly demonstrate that PknB of *M. bovis* BCG is in fact phosphorylated on Thr_309_.

Our analyses indicate that PknA is phosphorylated in at least one conserved residue, Ser_309_/Ser_310_ (see Table 2). Intriguingly, in this study *M. bovis* BCG PknA was found to be phosphorylated on seven different residues (three Ser and four Thr, respectively), all located in the juxtamembrane region. Unlike PknB, in PknA the juxtamembrane region, encompassing residue 269–338 is indispensable not only for auto-phosphorylation of PknA but also for its substrate phosphorylation ability (Thakur et al., 2008). STPks exhibit a wide variety of mechanisms for their regulation. Taking into account the degree of phosphorylation verified here in the juxtamembrane region of *M. bovis* BCG PknA compared to that observed in *M. smegmatis* PknA, it is tempting to speculate that this level of phosphorylation of the juxtamembrane region could be in fact limiting the access of PknA to its substrates and this way controlling the action of the enzyme. Importantly, as noted by Chawla et al. (2014), whilst the structure and mode of activation of PknB and PknA have been well established *in vitro*, the structure-function relationships of the various domains have yet to be investigated in the context of mycobacterial growth (Chawla et al., 2014). Here through a MS based phosphoproteomic approach we have established (at the phospho-site level) the phosphorylation state of different domains for both PknA and PknB *in vivo* during growth at exponential phase.

#### Conserved Tyr phosphorylated sites

**Figure 2:**
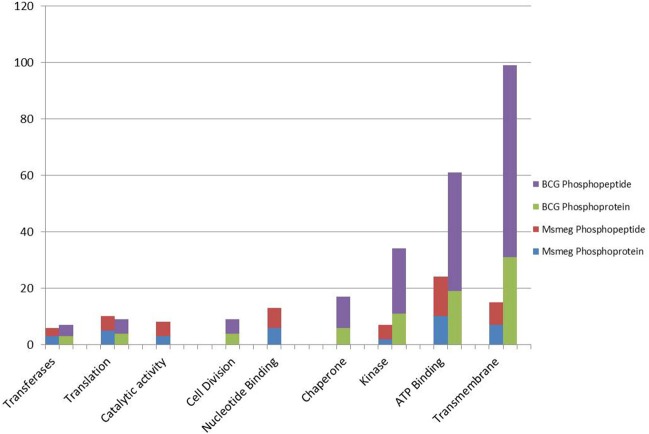
**A histogram showing the GO molecular functions of identified phosphoproteins and respective number of phosphopeptides as predicted from their genome annotations**.

In our study we have identified nine Tyr p-sites (see Table 1), of which four were also found to be phosphorylated in *M. tuberculosis* (Kusebauch et al., 2014): FHA-domain-containing protein (Tyr_215_ and Tyr_232_), 60 kDa chaperonin 1 (Tyr_358_) and conserved membrane protein mmpS3 (Tyr_70_) (Figure 1B). FHA-domain-containing protein is a substrate of numerous STPKs, including PknB. Phosphorylation of FHA by PnkB has implication in cell wall synthesis with a possible involvement in mycobacterial virulence (Gupta et al., 2009). Likewise both 60 kDa Chaperonin and conserved membrane protein mmpS3 have been implicated in mycobacterial virulence (Wells et al., 2013). Based on our data, we searched for possible conservation of these peptides across other bacterial species. A sequence motif derived from 60 kDa Chaperonin Tyr_358_ (RQEIEN**SDSDYDREKL**QERLA) using Seq2Logo revealed an overrepresentation of Tyr_358_ (Figure 2). A conserved Tyr_360_ residue on apparently conserved peptide (**SDSDYDREKL**) was found in three Gram negative pathogenic species, specifically *Shigella* spp., *Klebsiella* spp., and *Salmonella* spp., suggesting that this conserved Tyr phosphorylation site warrants further investigation for possible roles in bacterial pathogenesis.

**Figure 3:**
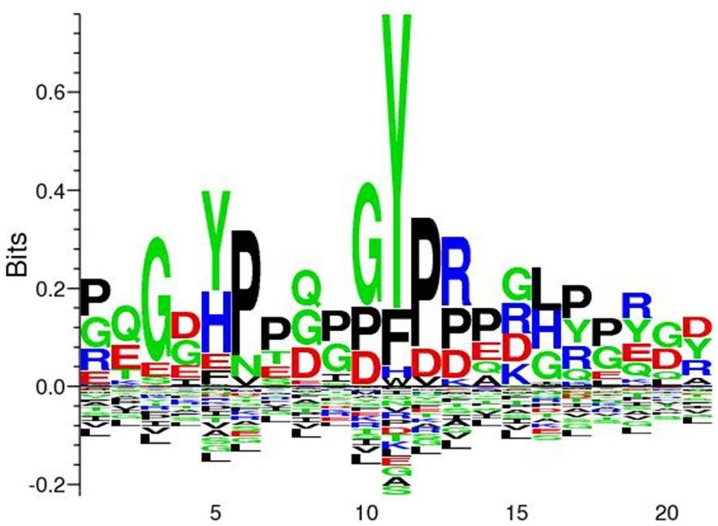
**Seq2Logo alignment analysis derived from 60 kDa chaperonin revelead an overrepresentation of Tyr_358_**. Seq2Logo analysis indicate that a conserved Tyr_358-360_ is found in additional three pathogenic species, specifically *Shigella* spp., *Klebsiella* spp., and *Salmonella* ssp.

### Phosphoprotein functional enrichment

Gene ontology (GO) terms revealed that in both *M. bovis* BCG and *M. smegmatis*, the phosphorproteins/phosphosites were functionally enriched in nine distinct groups, (e.g., ATP binding, translation, kinase activity, cell division, see Figure 2). Of interest, a great deal of phosphorylated proteins in *M. bovis* BCG was clustered into the Transmembrane group (Figure 2). This included a considerable number of multiple phosphorylated proteins and some phosphorylated in internal as well as external regions, like BCG_3967, it is a probable trans-membrane protein and we found it to be phosphorylated four times, at position 10, which like on the flagellin domain and position 801 and 801, the kinase domain. This suggests that there are transmembrane proteins with a potential role in signal transduction. Additionally, it was visible that *M bovis* BCG phosphoproteome comprised a notable group of phosphoproteins involved in cell division, possible implications of this is discussed below.

### Phosphorylation events observed in proteins that regulate mechanisms of growth and cell division

Both PknA and PknB are encoded by genes (*pkn*A and *pkn*B, respectively) located on the same operon as protein phosphatase PstP, RodA (implicated in cell shape control) and PbpA (implicated in peptidoglycan synthesis) (Cole et al., 1998). This locus includes also two FHA (forkhead-associated) domain-containing proteins and in mycobacteria is found near the origin of replication. In *M. bovis* BCG, all proteins referred to above (except PbpA) were found phosphorylated at a total of 15 p-sites: PknA (7); PknB (3); RodA (1); PstP (1) and FHA domain containing protein (3) (see Supplementary Table S1A). In *M. smegmatis*, however, only a few proteins were found phosphorylated at a total of 5 *p*-sites: PknA (2); PknB (2) FHA domain containing protein (1) (see Supplementary Table S1B). These observations suggest that the slow growth of *M. bovis* BCG preserves this central set of cell division proteins under a tight regulatory network in which key elements are intimately inter-related by an important series of functional (de)phosphorylation events. For example, PknA and PknB are regulated by PstP-mediated phosphorylation (Boitel et al., 2003; Chopra et al., 2003); additionally, recently it has been shown that both PknA and PknB phosphorylate PstP (Sajid et al., 2011). As discussed above, our results showed that both *M. smegmatis* and *M. bovis* BCG PknB has conserved phosphorylation on Thr_171_ and Thr_173_—both of which sites are known to be substrates for PstP—thus suggesting that in both cases PstP is at least partially inactive. This would make sense considering that during exponential phase PknA and PknB are likely to be at their peak of activity. PstP has been reported to be phosphorylated by PknB on Thr_173_, Thr_141_, Thr_290_, and Thr_137_ in its cytosolic domain and on Thr_174_ by PknA (Sajid et al., 2011). Curiously phosphorylated PstP has been reported to be more active than its unphosphorylated form (Sajid et al., 2011). Here, our results indicate that PstP of *M. bovis* BCG is phosphorylated *in vivo* on the high confidence *p*-site, Ser_155_ (see Supplementary Table S1A). Interestingly, PstP contains three metal-binding centers in its structure (Pullen et al., 2004), sharing the fold and two-metal center of human PP2Cα whilst having a third Mn^2+^ in a site created by a large shift in a flap domain next to the active site; this Mn^2+^ occurs at the position of Ser_160_ so it is plausible that phosphorylation of Ser_155_ may directly interfere with PstP activity, thus accounting for our deduction here of reduced PstP activity during exponential phase growth.

Overall, *M. bovis* BCG has 3 times more STPks and nearly 4 times respective *p*-sites compared to *M. smegmatis*. Apart from PknA and PknB, the *M. bovis* BCG phosphoproteome is comprised of PknG (Thr_95_), PknH (Thr_174_), PknE (Ser_304_ and Ser_326_) PknF (Thr_287_). Some of these enzymes have previously been directly implicated in mycobacterial growth [e.g., PknG (Fiuza et al., 2008), PknH (Zheng et al., 2007), PknE and PknF (Gupta et al., 2014)] and it is therefore conceivable that some of the proteins comprising the *M. bovis* BCG phosphoproteome are in fact substrate of some of these phosphorylated protein kinases (*vide infra*). It is worth noting that our study has also identified several Two Component sensory signal transduction proteins as phosphoproteins (e.g., Two component sensory transduction protein regX3 (Thr_151_ and Thr_153_), Two component sensor histidine kinase ppr (Ser_446_), Two component transcriptional regulatory pprA (Ser_20_). These results are reminiscient of those previously described in *B. subtilis* (Jers et al., 2011) and suggest that in *M bovis* BCG there may be cross talk between Ser/Thr/Tyr phosphorylation and Two component systems, which would add extra complexity to the overall protein phosphorylation signal transduction pathways regulating exponential growth of *M. bovis* BCG cells.

### Phosphorylation events observed in proteins that regulates mechanisms of cell elongation and division

In mycobacteria, cell elongation is regulated by a macrocomplex that regulates peptidoglycan remodeling during growth by means of hydrolytic and synthetic roles (as reviewed by (Kieser and Rubin, 2014)). Our data indicate that in *M. bovis* BCG, three important proteins of the macromolecular elongation complex are phosphorylated during exponential growth, namely Wag31 (Ser_245_), CwsA (Thr_77_) and a putative hydrolase (BCG_0021 involved in peptidoglycan catabolic process) (Thr_43_). In mycobacteria, Wag31 is phosphorylated by PknA and is essential for correct polar localization and biosynthesis (Jani et al., 2010; Lee et al., 2014); in addition, Wag31 is stabilized by the cell wall protein CwsA. Wag31 is thought to be phosphorylated during exponential phase and remains non- or lowly-phosphorylated during stationary phase (Kang et al., 2005; Park et al., 2008). Interestingly, Wag31, CwsA and the putative peptidoglycan hydrolase were not found amongst the *M. smegmatis* phosphorylated proteins in the present study. Whilst we cannot rule out that our assay did not isolate phosphorylated *M. smegmatis* Wag31, it is perhaps more likely that in the fast growing *M. smegmatis* the elongation complex is regulated by alternative non-phosphorylated mechanism.

Another macromolecular complex, named divisome, is responsible for mycobacterial cell division. Assembly and disassembly of this complex is regulated by protein phosphorylation (Kieser and Rubin, 2014). According to the our data, in *M. bovis* BCG there are five divisome proteins which are phosphorylated, including cell division FtsQ (Thr_24_), FtsW-like protein (Thr_29_), CwsA (Thr_77_) as well as other additional phosphorylated cell division proteins such as RodA (Thr_463_), cell division transmembrane protein FtsK (Thr_325_; Thr_642_) and FtsY (Thr_72_), strongly suggesting that divisome assembly and indeed cell division in *M. bovis* BCG is subject to a high level of phosphorylation.

Of interest, in our analysis we have detected Hup, a conserved histidine-like protein, phosphorylated on three different *p*-sites (Thr_45_, Thr_65_, and Ser_90_). In Mycobacterium sp., the homolog of HU (Mhpl) is implicated in bacterial adaptation to stress response conditions, possible inhibition of cellular metabolism and reduction of bacterial growth rate through nucleoid reorganization (Lee et al., 1998; Matsumoto et al., 1999; Katsube et al., 2007). Apparently, is expressed in exponentially growing cells of *M. tuberculosis* H37Ra and it is shown to be maximally expressed during stationary phase, while Hup kinases (PknE, PknF, and PknB) were found to be constitutively expressed during exponential phase (Gupta et al., 2014). It has been suggested that the phosphorylation of HupB during the exponential phase by the referred kinases would limit the interaction with DNA (Gupta et al., 2014). In our *M. bovis* BCG data we have identified all the intervenient proteins involved in the described posttranslational regulation mechanism, including the phosphorylation of phosphosite Hup Thr_65_. It is therefore appropriate to assume that the same mechanism takes place in *M. bovis* BCG cells during exponential growth, and although under limited action it remains possible that the rate of unphosphorylated HupB would have an impact on the overall growth rate. In contrast, we did not find any evidence to indicate that similar mechanisms operate in *M. smegmatis* cells during exponential growth.

### Stress related proteins

In rich broth during exponential phase, bacteria experience nearly optimal conditions of growth where there is excess nutrients and little accumulation of by products, in addition to scarce competition between bacterial cells. Surprisingly, under these conditions we observed an unexpected number of chaperones and stress related proteins in the *M. bovis* BCG phosphoproteome: For instance hyperosmotic and heat shock related proteins such as the chaperon protein DnaJ (Thr_120_) chaperon protein DnaK (Ser_558_) and GrpE (Ser_12_ and Thr_2_), multiply phosphorylated 60 kDa chaperonin, 10 kDa chaperonin, and Copper-sensing transcriptional repressor CsoR (Thr_93_). On the other hand none of these stress related proteins were found in the *M. smegmatis* phosphoproteome, which suggests that even under optimal environmental conditions, slow growing mycobacteria such as *M. bovis* BCG maintain a preventive basal level of stress-related proteins that may act as frontline defense barrier to ensure adequate and prompt response to any sudden change in local environmental conditions. In this scenario protein phosphorylation would keep most of these proteins in an inactive state, whereby dephosphorylation could then immediately recruit these proteins when environmental conditions become unfavorable. A convenient and versatile regulatory mechanism such as this could in fact be a determinant for the survival and persistence of some bacteria.

## Conclusion and prespectives

This study clearly demonstrated that there are major differences between a fast growing and a slow growing mycobacterial phosphoproteome. The *M. smegmatis* phosphoproteome observed here is in many aspects similar to those reported in other soil-dwelling bacterial models and can be viewed as a minimalist phosphoproteome compared to that of *M. bovis* BCG. This latter organism presents a much more complex and sophisticated protein phosphorylation network, regulating important cellular cycle events such as cell wall biosynthesis, elongation, and cell division, as well as apparently being involved in regulating response to stress, which over all would allow a quick cellular response to abrupt environmental changes. However, this regulatory advantage might be associated with a cost, reflected by reduced metabolic fitness and slower growth rate.

This study demonstrates *M. bovis* BCG is a good model to study aspects of mycobacterial phospho-dependent signal transduction pathways, including those involved in persistence and slow growth, including that associated with drug resistance. By contrast, the substantial differences reported here in the phosphoproteomes of *M. smegmatis* and *M. bovis* BCG suggest that exponentially growing *M. smegmatis* cells *in vitro* are of limited relevance when modeling phosphorylation networks and phospho-regulation events likely to occur in *M. tuberculosis* at the site of disease during an infection.

### Conflict of interest statement

The authors declare that the research was conducted in the absence of any commercial or financial relationships that could be construed as a potential conflict of interest.

## Supplementary material

Supplementary Figure S1 **Phosphoproteomic workflow diagram**. Mycobacterial strains were harvested at exponential phase, lysed. In-solution digestion was then carried out TiO_2_ phosphor enrichment and data acquisition with LC/MS/MS.

Supplementary Figure S2 **(a–f)**. Total Ion Chromatograms (TIC) and MS summary peptide/phosphopeptides identified in each experiment.

Supplementary S3A **Multiple sequence alignment of *M.bovis* BCG phosphoproteins with their respective *M. smegmatis***.

Supplementary S3B **Multiple sequence alignment of *M. smegmatis* phosphoproteins with their respective *M.bovis* BCG**.

Supplementary Table S1A **Spreadsheet of all the identified protein groups, phosphopeptides and phosphoproteins from *M. bovis* BCG**.

Supplementary Table S1B **Spreadsheet of all the identified protein groups, phosphopeptides and phosphoproteins from *M. smegmatis***.
